# Supplementary material for: Physical Activity and Sedentary Behavior Research in Indonesian Youth: A Scoping Review
Source: Int J Environ Res Public Health. 2020 Oct 21;17(20):7665. doi: 10.3390/ijerph17207665 (PMC7593924; doi:10.3390/ijerph17207665)
Supplement: Supplementary file 1 [file ijerph-17-07665-s001.zip › Table S2. Literature search strategy.docx]

Title: Physical activity and sedentary behavior research on Indonesian youth: A scoping review

Authors: Fitria Dwi Andriyani, Stuart J.H. Biddle, Novita Intan Arovah, Katrien De Cocker

Corresponding author: Fitria Dwi Andriyani, email: [FitriaDwi.Andriyani@usq.edu.au](mailto:FitriaDwi.Andriyani@usq.edu.au), [fitria.dwi.andriyani@uny.ac.id](mailto:fitria.dwi.andriyani@uny.ac.id)

**Table S2 (A) Literature search strategy (August 2018)**

| **Date of search** | **Search strategy** | **Database** | **Platform** | **Number of results** |
| --- | --- | --- | --- | --- |
| 10/8/2018 | (sedentary or inactive or sitting)-**All text** AND (TV or Television or video or computer or internet or gaming or "social media" or screen or "electronic media" or phone* or tablet or "play station") - **All text** AND (adolescen* or child* or school* or student* or youth or "young people") - **All text** AND (Indonesia* or Java or Sumatra or Kalimantan or Sulawesi or Bali or Maluku or Ambon or Papua or Nusa Tenggara) -**All text** | Academic Search Ultimate, CINAHL with Full Text, Education Research Complete, E-Journals, Health Source: Nursing/Academic Edition, MasterFILE Premier, PsycINFO, SPORTDiscus with Full Text | EBSCOhost Megafile ultimate | 125 |
| 10/8/2018 | ("physical activity" or "physical inactivity" or "physically inactive" or "physical fitness" or exercise or sport or "energy expenditure" or "motor activity" or walking or cycling or stair? or "active travel" or "active transport" or "strength training" "resistance training" or "weight training" or "weight lifting" or "muscle strengthening" or "muscle toning" or strength or toning or endurance) -**ALL TEXT** AND (adolescen* or child* or school* or student* or youth or "young people")-**All text**  AND (Indonesia* or Java or Sumatra or Kalimantan or Sulawesi or Bali or Maluku or Ambon or Papua or Nusa Tenggara)-**All text** | Academic Search Ultimate, CINAHL with Full Text, Education Research Complete, E-Journals, Health Source: Nursing/Academic Edition, MasterFILE Premier, PsycINFO, SPORTDiscus with Full Text | EBSCOhost Megafile ultimate | 122 |
| 13/8/2018 | (sedentary or inactive* or sitting) AND (TV or Television or video or computer or internet or gaming or "social media" or screen or "electronic media" or phone* or tablet or "play station") AND (adolescen* or child* or school* or student* or youth or "young people") AND (Indonesia* or Java or Sumatra or Kalimantan or Sulawesi or Bali or Maluku or Ambon or Papua or Nusa Tenggara) | Pubmed | Pubmed | 3 |
| 2/8/2018 | ("physical activity" or "physical inactivity" or "physically inactive" or "physical fitness" or exercise or sport or "energy expenditure" or "motor activity" or walking or cycling or stair? or "active travel" or "active transport" or "strength training" "resistance training" or "weight training" or "weight lifting" or "muscle strengthening" or "muscle toning" or strength or toning or endurance) AND (adolescen* or child* or school* or student* or youth or "young people") AND (Indonesia* or Java or Sumatra or Kalimantan or Sulawesi or Bali or Maluku or Ambon or Papua or Nusa Tenggara) | Pubmed | Pubmed | 43 |
| 10/8/2018 | (sedentary or inactiv* or sitting) **- Anywhere** AND (TV or Television or video or computer or internet or gaming or "social media" or screen or "electronic media" or phone* or tablet or "play station") **- Anywhere** AND (adolescen* or child* or school* or student* or youth or "young people") **- Anywhere** AND (Indonesia* or Java or Sumatra or Kalimantan or Sulawesi or Bali or Maluku or Ambon or Papua or Nusa Tenggara)**- Anywhere** |  | ProQuest dissertations and theses A&I. | 89 |
| 10/8/2018 | ("physical activity" or "physical inactivity" or "physically inactive" or "physical fitness" or exercise or sport or "energy expenditure" or "motor activity" or walking or cycling or stair? or "active travel" or "active transport" or "strength training" "resistance training" or "weight training" or "weight lifting" or "muscle strengthening" or "muscle toning" or strength or toning or endurance) -**Anywhere** AND (adolescen* or child* or school* or student* or youth or "young people") **- Anywhere** AND (Indonesia* or Java or Sumatra or Kalimantan or Sulawesi or Bali or Maluku or Ambon or Papua or Nusa Tenggara) -**Anywhere** |  | ProQuest dissertations and theses A&I. | 162 |
| 13/8/2018 | (sedentary or inactiv* or sitting) AND (adolescen* or child* or school* or student* or youth or "young people") AND (Indonesia*) |  | Google Scholar | 192 |
| 13/8/2018 | ("physical activity" or "physical inactivity" or "physically inactive" or "physical fitness") AND (adolescent* or child* or school* or student* or youth or "young people") AND (Indonesia*) |  | Google Scholar | 23 |
| 13/08/2018 | (Sedentary Or Sedentari) And (Remaja Or Anak Or Sekolah Or Siswa Or Murid Or Pelajar) And (Indonesia*) |  | Google Scholar | 320 |
| 13/08/2018 | (Sedentary Or Sedentari) And (Remaja Or Anak Or Sekolah Or Siswa Or Murid Or Pelajar) And (Indonesia*) |  | Google Scholar | 479 |
| 15/08/2018 | (Sedentary Or Sedentari) And (Remaja Or Anak Or Sekolah Or Siswa Or Murid Or Pelajar) And (Indonesia*) |  | Google Scholar | 599 |
| 15/08/2018 | ("Aktivitas Jasmani" Or " Kebugaran Jasmani" Or "Kesegaran Jasmani" Or "Perilaku Kurang Gerak" Or "Perilaku Tidak Aktif") And (Remaja Or Anak Or Sekolah Or Siswa Or Murid Or Pelajar) And (Indonesia*) |  | Google Scholar | 957 |
| 16/08/2018 | ("Aktivitas Jasmani" Or " Kebugaran Jasmani" Or "Kesegaran Jasmani" Or "Perilaku Kurang Gerak" Or "Perilaku Tidak Aktif") And (Remaja Or Anak Or Sekolah Or Siswa Or Murid Or Pelajar) And (Indonesia*) |  | Google Scholar | 880 |
| 16/08/2018 | ("Aktivitas Jasmani" Or " Kebugaran Jasmani" Or "Kesegaran Jasmani" Or "Perilaku Kurang Gerak" Or "Perilaku Tidak Aktif") And (Remaja Or Anak Or Sekolah Or Siswa Or Murid Or Pelajar) And (Indonesia*) |  | Google Scholar | 858 |
| 16/08/2018 | ("Aktivitas Jasmani" Or " Kebugaran Jasmani" Or "Kesegaran Jasmani" Or "Perilaku Kurang Gerak" Or "Perilaku Tidak Aktif") And (Remaja Or Anak Or Sekolah Or Siswa Or Murid Or Pelajar) And (Indonesia*) |  | Google Scholar | 800 |
| 17/08/2018 | ("Aktivitas Jasmani" Or " Kebugaran Jasmani" Or "Kesegaran Jasmani" Or "Perilaku Kurang Gerak" Or "Perilaku Tidak Aktif") And (Remaja Or Anak Or Sekolah Or Siswa Or Murid Or Pelajar) And (Indonesia*) |  | Google Scholar | 994 |
| 17/08/2018 | ("Aktivitas Jasmani" Or " Kebugaran Jasmani" Or "Kesegaran Jasmani" Or "Perilaku Kurang Gerak" Or "Perilaku Tidak Aktif") And (Remaja Or Anak Or Sekolah Or Siswa Or Murid Or Pelajar) And (Indonesia*) |  | Google Scholar | 994 |
| 17/08/2018 | ("Aktivitas Jasmani" Or " Kebugaran Jasmani" Or "Kesegaran Jasmani" Or "Perilaku Kurang Gerak" Or "Perilaku Tidak Aktif") And (Remaja Or Anak Or Sekolah Or Siswa Or Murid Or Pelajar) And (Indonesia*) |  | Google Scholar | 996 |
| 17/08/2018 | ("Aktivitas Jasmani" Or " Kebugaran Jasmani" Or "Kesegaran Jasmani" Or "Perilaku Kurang Gerak" Or "Perilaku Tidak Aktif") And (Remaja Or Anak Or Sekolah Or Siswa Or Murid Or Pelajar) And (Indonesia*) |  | Google Scholar | 697 |
| 6/8/2018 | RISKESDAS (National Basic Health Research) |  | Internet | 3 |
| 10/8/2018 | **TOPIC:**("physical activity" or "physical fitness" or exercise or sport or "energy expenditure" or "motor activity" or walking or cycling or stair? or "active travel" or "active transport" or "strength training" "resistance training" or "weight training" or "weight lifting" or "muscle strengthening" or "muscle toning" or strength or toning or endurance)) *AND* TOPIC: ((adolescen* or child* or school* or student* or youth or "young people")) *AND* TOPIC:((Indonesia* or Java or Sumatra or Kalimantan or Sulawesi or Bali or Maluku or Ambon or Papua or Nusa Tenggara) | Science Citation Index Expanded (SCI-EXPANDED) --1985-present, Social Sciences Citation Index (SSCI) --1985-present, Arts & Humanities Citation Index (A&HCI) --1985-present, Conference Proceedings Citation Index- Science (CPCI-S) --1990-present, Conference Proceedings Citation Index- Social Science & Humanities (CPCI-SSH) --1990-present, Emerging Sources Citation Index (ESCI) --2015-present | Web of Science | 197 |
| 10/8/2018 | TOPIC:((sedentary or inactiv* or sitting)) AND TOPIC: ((TV or Television or video or computer or internet or gaming or "social media" or screen or "electronic media" or phone* or tablet or "play station")) AND TOPIC:((adolescen* or child* or school* or student* or youth or "young people"))AND TOPIC: ((Indonesia* or Java or Sumatra or Kalimantan or Sulawesi or Bali or Maluku or Ambon or Papua or Nusa Tenggara)) | Science Citation Index Expanded (SCI-EXPANDED) --1985-present, Social Sciences Citation Index (SSCI) --1985-present, Arts & Humanities Citation Index (A&HCI) --1985-present, Conference Proceedings Citation Index- Science (CPCI-S) --1990-present, Conference Proceedings Citation Index- Social Science & Humanities (CPCI-SSH) --1990-present, Emerging Sources Citation Index (ESCI) --2015-present | Web of Science | 5 |
| 10/8/2018 | **TOPIC:**("physical activity" or "physical fitness" or exercise or sport or "energy expenditure" or "motor activity" or walking or cycling or stair? or "active travel" or "active transport" or "strength training" "resistance training" or "weight training" or "weight lifting" or "muscle strengthening" or "muscle toning" or strength or toning or endurance)) *AND* TOPIC: ((adolescen* or child* or school* or student* or youth or "young people")) *AND* TOPIC:((Indonesia* or Java or Sumatra or Kalimantan or Sulawesi or Bali or Maluku or Ambon or Papua or Nusa Tenggara) | MEDLINE | Web of Science | 188 |
| 10/8/2018 | TOPIC:((sedentary or inactiv* or sitting)) AND TOPIC: ((TV or Television or video or computer or internet or gaming or "social media" or screen or "electronic media" or phone* or tablet or "play station")) AND TOPIC:((adolescen* or child* or school* or student* or youth or "young people"))AND TOPIC: ((Indonesia* or Java or Sumatra or Kalimantan or Sulawesi or Bali or Maluku or Ambon or Papua or Nusa Tenggara)) | MEDLINE | Web of Science | 7 |
| 16/08/2018 | Aktivitas jasmani | Badan Penelitian dan Pengembangan Kesehatan, Kementerian Kesehatan Republik Indonesia (Balitbangkes Kemenkes) | neliti: Repositori Ilmiah Indonesia | 1 |
| 16/08/2018 | Kebugaran jasmani | Badan Penelitian dan Pengembangan Kesehatan, Kementerian Kesehatan Republik Indonesia (Balitbangkes Kemenkes) | neliti: Repositori Ilmiah Indonesia | 2 |
| 16/08/2018 | Sedentary | Badan Penelitian dan Pengembangan Kesehatan, Kementerian Kesehatan Republik Indonesia (Balitbangkes Kemenkes) | neliti: Repositori Ilmiah Indonesia | 5 |
| 16/08/2018 | Aktivitas jasmani, Kebugaran jasmani, sedentary, aktivitas fisik | Thesis S2 | Electronic Theses & Dissertations (ETD) Gadjah Mada University | 35 |
|  |  |  | **Total** | 9,776 |

**Table S2 (B). Literature search strategy (April 2020)**

| **Coverage of search** | **Search strategy** | **Database** | **Platform** | **Number of results** |
| --- | --- | --- | --- | --- |
| 10/08/2018 - 16/04/2020 | (sedentary or inactive or sitting)-**All text** AND (TV or Television or video or computer or internet or gaming or "social media" or screen or "electronic media" or phone* or tablet or "play station") - **All text** AND (adolescen* or child* or school* or student* or youth or "young people") - **All text** AND (Indonesia* or Java or Sumatra or Kalimantan or Sulawesi or Bali or Maluku or Ambon or Papua or Nusa Tenggara) -**All text** | Academic Search Ultimate, CINAHL with Full Text, Education Research Complete, E-Journals, Health Source: Nursing/Academic Edition, MasterFILE Premier, PsycINFO, SPORTDiscus with Full Text | EBSCOhost Megafile ultimate | 13 |
| 10/08/2018 - 16/04/2020 | ("physical activity" or "physical inactivity" or "physically inactive" or "physical fitness" or exercise or sport or "energy expenditure" or "motor activity" or walking or cycling or stair? or "active travel" or "active transport" or "strength training" "resistance training" or "weight training" or "weight lifting" or "muscle strengthening" or "muscle toning" or strength or toning or endurance) -**ALL TEXT** AND (adolescen* or child* or school* or student* or youth or "young people")-**All text**  AND (Indonesia* or Java or Sumatra or Kalimantan or Sulawesi or Bali or Maluku or Ambon or Papua or Nusa Tenggara)-**All text** | Academic Search Ultimate, CINAHL with Full Text, Education Research Complete, E-Journals, Health Source: Nursing/Academic Edition, MasterFILE Premier, PsycINFO, SPORTDiscus with Full Text | EBSCOhost Megafile ultimate | 8 |
| 2018-2020 | (sedentary or inactive* or sitting) AND (TV or Television or video or computer or internet or gaming or "social media" or screen or "electronic media" or phone* or tablet or "play station") AND (adolescen* or child* or school* or student* or youth or "young people") AND (Indonesia* or Java or Sumatra or Kalimantan or Sulawesi or Bali or Maluku or Ambon or Papua or Nusa Tenggara) | Pubmed | Pubmed | 3 |
| 2018-2020 | ("physical activity" or "physical inactivity" or "physically inactive" or "physical fitness" or exercise or sport or "energy expenditure" or "motor activity" or walking or cycling or stair? or "active travel" or "active transport" or "strength training" "resistance training" or "weight training" or "weight lifting" or "muscle strengthening" or "muscle toning" or strength or toning or endurance) AND (adolescen* or child* or school* or student* or youth or "young people") AND (Indonesia* or Java or Sumatra or Kalimantan or Sulawesi or Bali or Maluku or Ambon or Papua or Nusa Tenggara) | Pubmed | Pubmed | 82 |
| 2018-2020 | (sedentary or inactiv* or sitting) **- Anywhere** AND (TV or Television or video or computer or internet or gaming or "social media" or screen or "electronic media" or phone* or tablet or "play station") **- Anywhere** AND (adolescen* or child* or school* or student* or youth or "young people") **- Anywhere** AND (Indonesia* or Java or Sumatra or Kalimantan or Sulawesi or Bali or Maluku or Ambon or Papua or Nusa Tenggara)**- Anywhere** |  | ProQuest dissertations and theses A&I. | 2 |
| 2018-2020 | ("physical activity" or "physical inactivity" or "physically inactive" or "physical fitness" or exercise or sport or "energy expenditure" or "motor activity" or walking or cycling or stair? or "active travel" or "active transport" or "strength training" "resistance training" or "weight training" or "weight lifting" or "muscle strengthening" or "muscle toning" or strength or toning or endurance) -**Anywhere** AND (adolescen* or child* or school* or student* or youth or "young people") **- Anywhere** AND (Indonesia* or Java or Sumatra or Kalimantan or Sulawesi or Bali or Maluku or Ambon or Papua or Nusa Tenggara) -**Anywhere** |  | ProQuest dissertations and theses A&I. | 289 |
| 2018-2020 | (sedentary or inactiv* or sitting) AND (adolescen* or child* or school* or student* or youth or "young people") AND (Indonesia*) |  | Google Scholar | 49 |
| 2018-2020 | ("physical activity" or "physical inactivity" or "physically inactive" or "physical fitness") AND (adolescent* or child* or school* or student* or youth or "young people") AND (Indonesia*) |  | Google Scholar | 5 |
| 2018-2020 | (Sedentary Or Sedentari) And (Remaja Or Anak Or Sekolah Or Siswa Or Murid Or Pelajar) And (Indonesia*) |  | Google Scholar | 234 |
| 2018-2020 | ("Aktivitas Jasmani" Or " Kebugaran Jasmani" Or "Kesegaran Jasmani" Or "Perilaku Kurang Gerak" Or "Perilaku Tidak Aktif") And (Remaja Or Anak Or Sekolah Or Siswa Or Murid Or Pelajar) And (Indonesia*) |  | Google Scholar | 175 |
| 8/06/2018 | RISKESDAS (National Basic Health Research) 2018 |  | Google | 1 |
| 2018-2020 | **TOPIC:**("physical activity" or "physical fitness" or exercise or sport or "energy expenditure" or "motor activity" or walking or cycling or stair? or "active travel" or "active transport" or "strength training" "resistance training" or "weight training" or "weight lifting" or "muscle strengthening" or "muscle toning" or strength or toning or endurance)) *AND* TOPIC: ((adolescen* or child* or school* or student* or youth or "young people")) *AND* TOPIC:((Indonesia* or Java or Sumatra or Kalimantan or Sulawesi or Bali or Maluku or Ambon or Papua or Nusa Tenggara) | Science Citation Index Expanded (SCI-EXPANDED) --1985-present, Social Sciences Citation Index (SSCI) --1985-present, Arts & Humanities Citation Index (A&HCI) --1985-present, Conference Proceedings Citation Index- Science (CPCI-S) --1990-present, Conference Proceedings Citation Index- Social Science & Humanities (CPCI-SSH) --1990-present, Emerging Sources Citation Index (ESCI) --2015-present | Web of Science | 113 |
| 2018-2020 | TOPIC:((sedentary or inactiv* or sitting)) AND TOPIC: ((TV or Television or video or computer or internet or gaming or "social media" or screen or "electronic media" or phone* or tablet or "play station")) AND TOPIC:((adolescen* or child* or school* or student* or youth or "young people"))AND TOPIC: ((Indonesia* or Java or Sumatra or Kalimantan or Sulawesi or Bali or Maluku or Ambon or Papua or Nusa Tenggara)) | Science Citation Index Expanded (SCI-EXPANDED) --1985-present, Social Sciences Citation Index (SSCI) --1985-present, Arts & Humanities Citation Index (A&HCI) --1985-present, Conference Proceedings Citation Index- Science (CPCI-S) --1990-present, Conference Proceedings Citation Index- Social Science & Humanities (CPCI-SSH) --1990-present, Emerging Sources Citation Index (ESCI) --2015-present | Web of Science | 2 |
| 2018-2020 | **TOPIC:**("physical activity" or "physical fitness" or exercise or sport or "energy expenditure" or "motor activity" or walking or cycling or stair? or "active travel" or "active transport" or "strength training" "resistance training" or "weight training" or "weight lifting" or "muscle strengthening" or "muscle toning" or strength or toning or endurance)) *AND* TOPIC: ((adolescen* or child* or school* or student* or youth or "young people")) *AND* TOPIC:((Indonesia* or Java or Sumatra or Kalimantan or Sulawesi or Bali or Maluku or Ambon or Papua or Nusa Tenggara) | MEDLINE | Web of Science | 30 |
| 2018-2020 | TOPIC:((sedentary or inactiv* or sitting)) AND TOPIC:((adolescen* or child* or school* or student* or youth or "young people"))AND TOPIC: ((Indonesia* or Java or Sumatra or Kalimantan or Sulawesi or Bali or Maluku or Ambon or Papua or Nusa Tenggara)) | MEDLINE | Web of Science | 5 |
| 16/04/2020 | Aktivitas jasmani | Badan Penelitian dan Pengembangan Kesehatan, Kementerian Kesehatan Republik Indonesia (Balitbangkes Kemenkes) | neliti: Repositori Ilmiah Indonesia | 0 |
| 16/04/2020 | Kebugaran jasmani | Badan Penelitian dan Pengembangan Kesehatan, Kementerian Kesehatan Republik Indonesia (Balitbangkes Kemenkes) | neliti: Repositori Ilmiah Indonesia | 0 |
| 16/04/2020 | Sedentary | Badan Penelitian dan Pengembangan Kesehatan, Kementerian Kesehatan Republik Indonesia (Balitbangkes Kemenkes) | neliti: Repositori Ilmiah Indonesia | 0 |
| 16/04/2020 | Aktivitas jasmani, Kebugaran jasmani, sedentary, aktivitas fisik | Thesis S2 | Electronic Theses & Dissertations (ETD) Gadjah Mada University | 12 |
|  |  |  | **Total** | 1,023 |
