# Supplementary material for: Physical Activity and Sedentary Behavior Research in Indonesian Youth: A Scoping Review
Source: Int J Environ Res Public Health. 2020 Oct 21;17(20):7665. doi: 10.3390/ijerph17207665 (PMC7593924; doi:10.3390/ijerph17207665)
Supplement: Supplementary file 1 [file ijerph-17-07665-s001.zip › Table S4. Study topic and subtopic of included studies.docx]

Title: Physical activity and sedentary behavior research on Indonesian youth: A scoping review

Authors: Fitria Dwi Andriyani, Stuart J.H. Biddle, Novita Intan Arovah, Katrien De Cocker

Corresponding author: Fitria Dwi Andriyani, email: [FitriaDwi.Andriyani@usq.edu.au](mailto:FitriaDwi.Andriyani@usq.edu.au), [fitria.dwi.andriyani@uny.ac.id](mailto:fitria.dwi.andriyani@uny.ac.id)

**Table S4. Study topic and subtopic of included studies**

| Topic and Subtopic | n | % |
| --- | --- | --- |
| Study topic |  |  |
| PA only | 112 | 67.5 |
| SB only | 25 | 15 |
| PA and SB (Both) | 29 | 17.5 |
| Total | 166 | 100.0 |
| Study subtopic |  |  |
| Correlates of PA | 71 | 24.6 |
| Correlates of SB | 37 | 12.8 |
| Outcomes of PA | 49 | 17.0 |
| Outcomes of SB | 1 | 0.3 |
| Includes Prevalence or measurement of PA | 81 | 28.0 |
| Includes Prevalence or measurement of SB | 46 | 15.9 |
| Prevalence study of PA | 2 | 0.7 |
| Prevalence study of SB | 1 | 0.3 |
| Validation study | 1 | 0.3 |
| Total * | 289 | 100.0 |
| Note: *Multiple correlates and/or outcomes were investigated in some studies; hence the sum of the totals is greater than the total number of included studies. PA: physical activity; SB: sedentary behavior | | |
