# Supplementary material for: Physical Activity and Sedentary Behavior Research in Indonesian Youth: A Scoping Review
Source: Int J Environ Res Public Health. 2020 Oct 21;17(20):7665. doi: 10.3390/ijerph17207665 (PMC7593924; doi:10.3390/ijerph17207665)
Supplement: Supplementary file 1 [file ijerph-17-07665-s001.zip › Table S5. Summary of research design.docx]

Title: Physical activity and sedentary behavior research on Indonesian children and adolescents: A scoping review

Authors: Fitria Dwi Andriyani, Stuart J.H. Biddle, Novita Intan Arovah, Katrien De Cocker

Corresponding author: Fitria Dwi Andriyani, email: [FitriaDwi.Andriyani@usq.edu.au](mailto:FitriaDwi.Andriyani@usq.edu.au), [fitria.dwi.andriyani@uny.ac.id](mailto:fitria.dwi.andriyani@uny.ac.id)

**Table S5. The Summary of Research Design**

| Methods | Number of studies | % | |
| --- | --- | --- | --- |
| Design |  |  | |
| Retrospective cohort study | 1 | 0.6 | |
| Intervention | 50 | 30.1 | |
| Case control | 22 | 13.3 | |
| Cross sectional | 93 | 56.0 | |
| Total | 166 | 100 | |
| Approach |  |  | |
| Quantitative | 164 | 98.8 | |
| Qualitative | 0 | 0 | |
| Mixed-methods | 2 | 1.2 | |
| Total | 166 | 100 | |
| Sampling |  |  | |
| Random sampling | 37 | 22.3 | |
| Purposive sampling | 47 | 28.3 | |
| Consecutive sampling | 9 | 5.4 | |
| Cluster sampling | 12 | 7.2 | |
| Proportional to size | 2 | 1.2 | |
| Quota sampling | 1 | 0.6 | |
| Convenience sampling | 2 | 1.2 | |
| Accidental sampling | 1 | 0.6 | |
| Total sampling | 8 | 4.8 | |
| Multiple sampling methods | 28 | 16.9 | |
| Not specified | 19 | 11.4 | |
| Total | 166 | 100 | |
| Measurement Method |  |  | |
| Self-administered | 49 | 29.3 | |
| Interview-administered | 23 | 13.8 | |
| Proxy report | 5 | 3.0 | |
| Test | 36 | 21.6 | |
| Measurement | 5 | 3.0 | |
| Device-based | 4 | 2.4 | |
| Observation | 1 | 0.6 | |
| Not specified | 44 | 26.3 | |
| Total* | 167 | 100 | |
| Note: *Two different methods were used in one study; hence the sum of the totals is higher than the total number of included studies | | |  |
