# Supplementary material for: Physical Activity and Sedentary Behavior Research in Indonesian Youth: A Scoping Review
Source: Int J Environ Res Public Health. 2020 Oct 21;17(20):7665. doi: 10.3390/ijerph17207665 (PMC7593924; doi:10.3390/ijerph17207665)
Supplement: Supplementary file 1 [file ijerph-17-07665-s001.zip › Table S6. Characteristics of study samples (MS Word).docx]

Title: Physical activity and sedentary behavior research on Indonesian children and adolescents: A scoping review

Authors: Fitria Dwi Andriyani, Stuart J.H. Biddle, Novita Intan Arovah, Katrien De Cocker

Corresponding author: Fitria Dwi Andriyani, email: [FitriaDwi.Andriyani@usq.edu.au](mailto:FitriaDwi.Andriyani@usq.edu.au), [fitria.dwi.andriyani@uny.ac.id](mailto:fitria.dwi.andriyani@uny.ac.id)

**Table S6. Characteristics of study samples**

|  | Number of study | % |
| --- | --- | --- |
| Sex |  |  |
| Female | 15 | 9.0 |
| Male | 10 | 6.0 |
| Both | 131 | 79.0 |
| Not specified | 10 | 6.0 |
| Total | 166 | 100% |
| Geographical type |  |  |
| Rural | 6 | 3.6 |
| Urban | 7 | 4.2 |
| Both | 13 | 7.8 |
| Not specified | 140 | 84.3 |
| Total | 166 | 100% |
| Island (Area) |  |  |
| Java | 102 | 61.4 |
| Sumatera | 11 | 6.6 |
| Sulawesi | 10 | 6.0 |
| Kalimantan | 8 | 4.8 |
| Bali | 8 | 4.8 |
| Papua | 2 | 1.2 |
| Multi-islands | 2 | 1.2 |
| Indonesia | 20 | 12.0 |
| Not specified | 3 | 1.8 |
| Total | 166 | 100% |
| Population group/school level |  |  |
| Primary school | 55 | 33.1 |
| Junior high school | 33 | 19.9 |
| Senior high school | 49 | 29.5 |
| Multi-level school | 6 | 3.6 |
| Adolescents | 11 | 6.6 |
| University | 1 | 0.6 |
| Not specified | 11 | 6.6 |
| Total | 166 | 100% |
